# Performance of integrated sequencing batch reactor (SBR) and reverse osmosis (RO) process for leachate treatment: effect of pH

**DOI:** 10.1007/s40201-022-00788-0

**Published:** 2022-01-12

**Authors:** Izabela Anna Tałałaj

**Affiliations:** Faculty of Civil Engineering and Environmental Sciences, Bialystok University of Technology, Wiejska 45E Street, 15-351 Bialystok, Poland

**Keywords:** Landfill, Leachate, RO performance, pH, Reverse osmosis

## Abstract

**Purpose:**

In this paper the performance and effectiveness of the reverse osmosis (RO) process for the biologically pretreated leachate was investigated. The RO process was carried out separately for two different pH: 8.0 and 9.3.

**Methods:**

A general pollution parameters as well as organic and inorganic indicators were determined in raw, biologically pretreated and RO treated leachate. The performance characteristics of the reverse osmosis system were made on the basis of permeate flux, electroconductivity removal rate, concentration factor and efficiency in removal of analyzed parameters.

**Results:**

The use of SBR pretreatment had very good efficiency in BOD (97.3%) and ammonia nitrogen (95.4%) removal. The lowest effectivity was observed for chloride (11.6%), boron (3.9%) and TDS (1.2%). Pretreated leachate was subjected to RO system. The normalized average flux was 0.53 (42.3 L/m^2^·h) for pH = 8.0 and 0.68 (33.5 L/m^2^·h) for pH = 9.3. The lower membrane fouling at higher pH can be explained by electrostatic repulsion between the negatively charged membrane surface and organic substances. Independently of the process pH, a two-step membrane fouling was observed. The greatest differences in removal rates were observed for boron, which had a higher retention rate at higher pH, and ammonia nitrogen, whose removal rate decreased at higher pH. The obtained permeate pH after RO process was lower than the feed pH in two analyzed value of pH.

**Conclusions:**

The higher flux value at pH = 9.3 is result of high content of organic matter in leachate, which is better rejected at higher pH because of higher electrostatic repulsion between organic matter and membrane surface. This indicates that the organic matter content should be taken into account when determining the operating parameters (pH values) of the RO system.

## Introduction

Landfilling is still the commonly used method for final disposal of municipal solid waste [1, 2]. One of the main environmental problems arising during solid waste landfilling is the generation of heavily polluted landfill leachates [3]. They are highly loaded wastewater containing a wide variety of pollutants, including organic compounds, heavy metals and inorganic salts [1, 3–5]. The concentration of most components in landfill leachate exceed the concentration of the same in the sewage sludge [2].

In recent years, different technologies, including biological and physicochemical treatment have been used for leachate treatment [1, 3, 6, 7]. Biological methods give quite satisfactory performance when used for leachate from young landfill with less than 10 years of exploitation. They are effective for removal of organic substances, suspended solids and nutrients [3, 8, 9]. This method is commonly used to treat landfill leachate containing high value of Biological Oxygen Demand (BOD) [1, 10]. However, when treating stabilized leachate from an old landfill sites, biological treatment may not be enough to achieve the permitted discharge limits, due to high quantity of refractory organic compounds, which are not biodegradable under normal conditions, and a high percentage of the total nitrogen existing as a ammonium anions [2, 7, 11]. Moreover, a significant amount of inorganic salts, that do not undergo to biological treatment are present in leachate [12]. So, in order to achieve high treatment efficiency, a combination of biological and physico-chemical method is used for treatment landfill leachate.

With the development in leachate treatment technology, the high pressure membrane processes, like reverse osmosis have been employed intensively to complete biological methods. The use of such an integrated system of leachate treatment is also favorable for the RO performance, because providing an effective pretreatment of landfill leachate is the most effective solution of membrane fouling prevention. Fouling is associated with the accumulation of different kind particles on membrane surface or within the membrane pores. It can limit membrane process efficiency, as it lead to flux decline uneconomical increase in applied pressure and the need to frequent cleaning. Nevertheless, a reverse osmosis process is a complex process, in which the ionic transport mechanism and selectivity of membrane depends on many factors, among which are: charge (Donnan effect), steric and dielectric effects as well as membrane characteristics. The nature of the membrane and electrolytes are responsible for the charge polarities between membrane and the solutes, the steric effect is caused by the relative size of ions to the membrane pores, and the dielectric effect is caused by the differences in dielectric constant between bulk and membrane pores. These effects complement the abilities of the membrane used in separation process [13].

Several studies were performed to investigate the performance of RO with biological pretreated leachate. A combination of membrane biological reactor (MBR) and RO was studied by Ahmed and Lan [14] for matured landfill leachate. Authors used MBR for decrease the value of BOD in influent as well as for removal of N-NH_4_^+^ and suspended solids, which aids in minimalizing clogging and fouling problems during RO process. A combination of MBR and RO have given 97% removal of chemical oxygen demand (COD) and 96% removal of N-NH_4_^+^. Similar result obtained Hasar et al. [15] during treatment of leachate with BOD/COD ratio in the range from 0.4 to 0.7. Authors pointed high COD removal and over 99% decrease of electroconductivity (EC) value. Also, MBR which consist of MF/UF and membrane bioreactor containing activated sludge, is used in the leachate treatment process [16–18]. Its advantages are: good process stability, good effluent quality and low sludge production [19]. Nevertheless, according to Keyikoglu [20] MF/UF membranes are not as efficient as the RO ones, especially in the removal of humic acids, proteins as well as divalent and monovalent ions. A combination of activated sludge (AS) and RO was undertaken by Li et al. [21]. About 63% of COD removal was achieved in AS effluent, and over than 99% after AC and RO process. The reduction rate after the two stage treatment was also high for dissolved solids (98%), chloride (99%) and N-NH_4_^+^ (98%). Bohdziewicz and Kwarciak [22] showed an effective removal of leachate contaminants by using reverse osmosis following up-flow anaerobic sludge blanket (UASB). Removal of COD after UASB achieved the value of 76%. Due to poor quality of UASB effluent leachate was put into RO post-treatment process. After reverse osmosis filtration value of COD, BOD and N-NH_4_^+^ were removed in 95.4%, 90.2%, 88.7%, respectively [22].

From the literature review it appears, that many studies have considered the efficiency of biological methods integrated with RO. Most of them focused on selected biological methods such as UASB or MBR. There are only few studies concerning treatment of landfill leachate with sequencing batch reactors (SBR) process as a pretreatment. That is why for analyses of leachate treatment effectiveness, a combination of SBR + RO system was used. The advantage of SBR is process flexibility, which is desirable when considering landfill leachate with significant variations in quality and quantity. Moreover, it tolerates shock load caused by organic and hydraulic load variability. The system is easily configured and adjusted for short-term diurnal and long-term seasonal variations [23]. Another advantages of SBR is being easier to prevent filamentous sludge bulking settling problems [24]. According to Keyikoglu et al. [20] use of integrated SBR + RO process can give a good results in nitrification rate and organic matter removal as well as elimination of non-biodegradable matter and inorganic nitrogenous ions.

Most authors have investigated the effectiveness of reverse osmosis for leachate treatment in pH = 6.5 [9, 25–27]. Acidification of leachate to this value increases the solubility of the salts and prevents scaling phenomenon. Scaling occurs when the concentration of sparingly soluble inorganic salts such as calcium sulfates and carbonates increase above the saturation level. As the result, these salts precipitate and deposit on membrane. The scaling causes a decline in flux and reduction in membrane life [28, 29]. However, for leachate containing organic matter, acidification can increase organic fouling leading to decrease in permeate flow. Some authors have found that for leachate containing organic matter, fouling can be reduced by filtration process conducted at pH = 8, but only a few ones have used this pH value for leachate treatment [30, 31]. Value pH of 8.0 favors the deprotonation of H + ions from humic substances present in the leachate and makes membrane charge more negatively than at lower values of pH. This causes an increase in electrostatic repulsion between organic matter and membrane, limiting the fouling phenomenon [31–33].

Considering the above information, the aim of this paper is performance analysis of integrated SBR + RO system for leachate treatment at two variants: without pH correction after biological pretreatment (pH = 9.3) and with correction to pH of 8.0.

## Material and methods

### Material

The leachate samples used in this work were from municipal landfill located in north-western Poland. The landfill is operated since 1983 and occupies an area of 25.5 ha with maximum daily intake capacity of 10 Mg of waste. The landfill consists of two cells: Cell A – an old cell exploited since 1983 and closed in 2011, and Cell B – working since 2012. The leachate samples were collected from exploited part of landfill – Cell B, directly from the manhole on the drainage system. Collected samples were stored at 4 °C, in dark place and analyzed within one day.

### SBR pretreatment

A sequencing batch reactor (SBR) with a total volume of 25 L were used for the landfill pretreatment before reverse osmosis process. The biological process was realized with reactor filled with 15 L of leachate. The reactor was equipped with a mechanical stirrer to ensure complete mixing. Aeration was carried out by air diffusers placed at the bottom part of the reactor. The biological reactor was fed with activated sludge collected at a municipal sewage treatment plant. The activated sludge was adapted for the time of six weeks to the contaminants present in leachate, and then the main test were started.

A complete working cycle of the SBR (with 20.5 h total operation time) included following phases: mixing/denitrification (120 min), mixing and aeration/nitrification (240 min), external carbon dosage (3 cm^3^) mixing/denitrification (180 min), mixing and aeration/nitrification (240 min), external carbon dosage (3 cm^3^) mixing/denitrification (180 min), mixing and aeration/nitrification (240 min), settling (30 min), decantation (of 1.7 L) and withdraw (of 0.3 L). The SBR process was led with sludge concentration of 6 g/L.

After a full SBR cycle the pretreated leachate was directed to physico–chemical analyses. The pH measured for leachate after SBR pretreatment was 9.3.

### RO treatment

The reverse osmosis process was carried out separately for two different leachate pH: pretreated leachate with pH = 9.3 and pretreated leachate with corrected pH to the value of 8.0 (hydrochloric acid was used for pH adjustment).

The reverse osmosis process was conducted using laboratory RO module RO20NS.1, which was designed and constructed to reflect the conditions of reverse osmosis units operating in municipal landfills. According to manufacturer’s recommendations the RO module can be used for separation/concentration and for evaluation of this process in leachate treatment. The basic component of the system was Micro 240 PCI membrane module equipped with two tubular membranes with total area of 240 cm^2^. The system was also equipped with a pump pulsation damper, a flow meter, safety valve and two pressure gauges enabling pressure measurement on the liquid entry side to the system and to control the RO process pressure. Before entering the RO system, landfill leachate were subjected to filtration successively on the 50 and 5 μm filters.

**Fig. 1:**
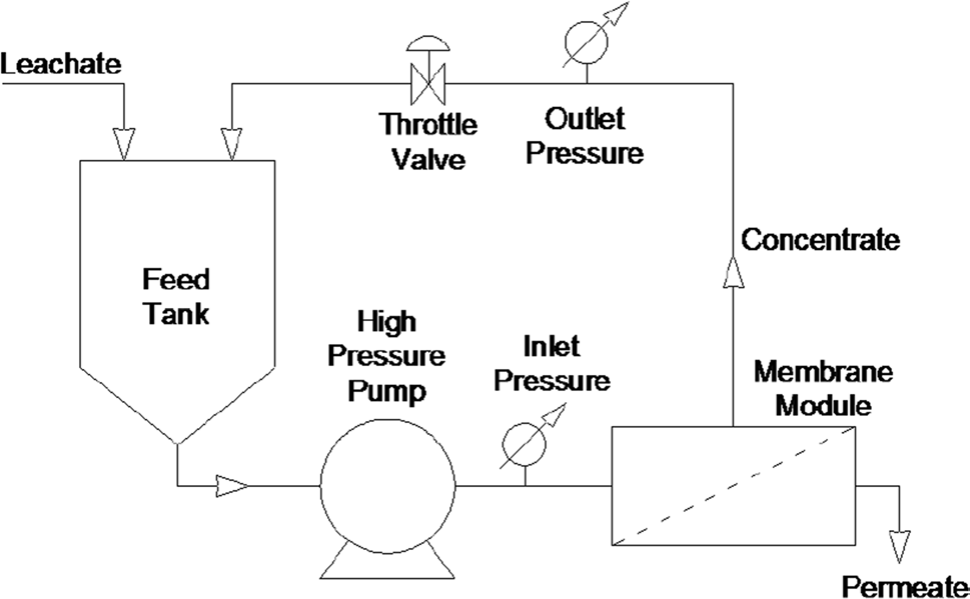
The reverse osmosis installation

The process was carried on with concentrate recirculation to the feed tank supplying the inlet to the RO system, i.e. in the same way, as when operating in landfills. The recovery rate was set at 60/40 (60% permeate and 40% concentrate) so that it also replicates real working conditions at landfills. The RO was operated in cross-flow filtration mode with the feed rate of 18 dm^3^/min, temperature of 25 °C and stable pressure of 3.8 MPa. Parameters of system operations were monitored periodically at 30 min intervals. The initial flux was determined on the base of permeate volume generated during first 30 min of RO filtration/operation. The separation process was carried out until the assumed 60% recovery rate was achieved. The schema of research installation is given in Fig. 1.

RO tubular membranes made of polyamide, obtained from PCI Membranes, were used in experiment. New membranes were washed and soaked in deionized water for 24 h and recirculated for 30 min before use.

After every RO process filtration, a membrane cleaning was run. According to the manufacturer's instruction, a solution of 0.25% (by weight) of Ultrasil 11 was used, and membranes were washed for 45 min at temperature of 50 °C. Cleaning of osmotic membranes was carried out at a pressure of 1.5 MPa. After cleaning, its effectiveness was assessed by measuring the value of permeate flux (m^3^/m^2^⋅s) and calculating of permeability lost. Reverse osmosis specification and membrane properties is presented in Table 1 [34, 35].

**Table 1:** RO membrane characteristics

RO module specification	
Permeate flow	5 ÷ 50 ml/min
Pressure	max 5.0 MPa
Recycle flow rate	max. 20 dm^3^/min
Tubeside volume	750 ml
Dimension of membrane used in RO module	2 membranes with 1.25 cm dia × 30 cm tubes
Membrane area	240 cm^2^ (0,024 m^2^)
Membrane material	
Membrane area	240 cm^2^ (0,024 m^2^)
Membrane material	Polyamide film
pH range	1.5 ÷ 12
Operating pressure	6.4 MPa
Operating temperature	80 °C
Nominal retention character	99% NaCl
hydrophilicity	3^*^
Solvent resistance	2^**^
Isoelectric point (pH)	about 4
Contact angle (°)	40 ÷ 63

^*^ 1 Low, 5 high

^**^1 Low, 3 high

The performance characteristics of the reverse osmosis system were made on the basis of the value of: permeate flux, removal rate of salts, concentration factor of EC in concentrate and removal ratio. The permeate flux (J) was calculated according the formula [36]:

1 $$J_{{}} = k \cdot (\frac{{V_{p} }}{t \cdot S})\left( {{\text{L}}/{\text{m}}^{{2}} \cdot{\text{h}}} \right)$$

where: V_p_—permeate volume (L), $t$—time (h), S- membrane area (m^2^), k – temperature correction factor.

The normalized flux were calculated as a J/J_o_ ratio, where J_0_ was the initial permeate flux determined on the base of permeate volume generated during the first 30 min of RO filtration/operation and J is permeate flux at a given time.

The removal (retention) rate of salts expressed as a electroconductivity value (R_EC_) was calculated using the following equation [36, 37]:

2 $$R_{EC} = (1 - \frac{{EC_{p} }}{{EC_{n} }}) \cdot 100\% \left( \% \right)$$

where: EC_p_ – electroconductivity of permeate (mS/cm), EC_n_ – electroconductivity of inlet (leachate) (mS/cm).

For calculation of concentration factor of EC in concentrate (CF_EC_) a following formula was used [36, 37]:

3 $$CF_{EC} = \frac{{EC_{c} }}{{EC_{n} }}$$

where: EC_c_ – electroconductivity of concentrate (mS/cm).

To assess the effectiveness of RO a removal ratio (R) was calculated for each contaminant [36, 37]:

4 $$\mathrm{R}=(1-\frac{{\mathrm{c}}_{\mathrm{p}}}{{\mathrm{C}}_{\mathrm{n}}})\cdot 100\mathrm{\%}$$

where: C_p_ –contaminant concentration of permeate (mg/L), C_n_ – contaminant concentration of inlet (leachate) (mg/L).

### Analytical methods

In raw, pretreated and treated leachate three groups of parameters were determined: a) general parameters such as the pH, EC, TDS; b) organic indicators such as the BOD, COD; c) inorganic parameters such as N-NH_4_^+^, Cl^−^ and boron. For leachate after SBR pretreatment the concentration of N-NO_3_^−^ was also analyzed. All analyses were made according to the Standard Methods [38].

The electroconductivity and the pH were measured on-site by a conductivity and potentiometric method, respectively, using a portable pH/conductivity meter (HACH HQ40d). Total dissolved solids were determined by a mass balance method after a well-mixed sample filtration through a FILTRAK cellulose fiber filter, and the residue retained on the filter was dried for a 1 h at 103-105^0^C in drying oven (to constant weight). The chemical oxygen demand was analyzed using a calorimetric method with a HACH spectrophotometer (620 nm) after a 2-h reactor digestion (a K_2_Cr_2_O_7_ method) and the biochemical oxygen demand – using an OxiTop (WTW) measuring system based on a pressure measurement. Concentration of nitrogen ammonia and nitrate nitrogen were analyzed with N Tube Vials on the HACH spectrophotometer (655 nm, 345 nm) using a salicylate method for N-NH_4_^+^ and Dimethylphenol Method for N-NO_3_^−^. Chloride was analyzed by an iron(III)-thiocyanade method (468 nm), boron was measured by its reaction with carminic acid in the presence of sulphuric acid (605 nm). Analyses were performed with the use of the HACH DR2000 and DR 3900 spectrophotometers.

## Results and discussion

### Leachate characteristic

The result of raw and biologically pretreated leachate characteristic is presented in Table 2. Fresh leachate was alkaline with a strong dark color associated with high organic loading (COD = 3720 mg/L, BOD = 690 mg/L). The leachate characterized with high conductivity of 17.9 mS/cm, which was attributed to the high concentration of dissolved solids such as: chloride, ammonium, sulphate, boron etc. The sample also contained a high content of TDS with value of 10,800 mg/L. The concentrations of raw leachate were found within reported range and suggested that analyzed leachate are at intermediate stage, between acidogenic and matured (methanogenic) phase of biodegradation [3, 39–42].

**Table 2:** Raw and biologically pretreated leachate characteristic

Parameter	Unit	Raw leachate	SBR removal efficiency ± st.dev (%)
pH	-	7.7	-
EC	mS/cm	17.9	37.2 ± 0.8
TDS	mg/L	10,800	1.2 ± 0.7
COD	mg O_2_/L	3720	32.5 ± 0.6
BOD	mg O_2_/L	690	97.2 ± 0.2
N-NH_4_^+^	mg/L	910	95.4 ± 0.3
N-NO_3_^−^	mg/L	1.59	> 99.9 ± 0.1
B	mg/L	12.7	3.9 ± 0.3
Cl^−^	mg/L	2490	11.6 ± 0.1

st.dev-standard deviation.

Table 2 also shows the characteristic of leachate pretreated in sequencing batch reactor. It was found that biological pretreatment effectively reduced mainly biodegradable organic substances expressed as a BOD (97.2%) and in large part removed nitrogen ammonia (95.4%) from leachate. The biological pretreatment was moderately effective in lowering the value of EC (37.2%) and recalcitrant organic substances expressed as a COD (32.5%). It is in agreement with Trebouet et al. [43] which says that leachate from biological stage still shows high COD values because the fulvic-like fraction with molecular-weight (MW) from 500 to 1000 increases after biological treatment. The removal efficiency of chloride, boron and TDS was 11.6%, 3.9% and 1.2%, respectively. The pH of leachate after SBR pretreatment was 9.3 and was a result of high nitrate reduction (denitrification). According to Kaszubowska [44] in the denitrification process, the alkalinity increases with the loss of nitrate nitrogen by about 3 g CaCO3/a NO3-N. Biologically pretreated leachate was directed to the RO system for further treatment process.

### Reverse osmosis performance

The reverse osmosis process for biologically treated leachate was carried out in two variants. In the first variant, SBR effluents were directed to the RO system without pH correction (i.e. with pH of 9.3). In the second variant, the pH of SBR effluent was adjusted to 8.0 before the RO process with use of hydrochloric acid. For each pH variant, a sample of leachate with a volume of 6 L was subjected to filtration.

- Variation of flux

**Fig. 2:**
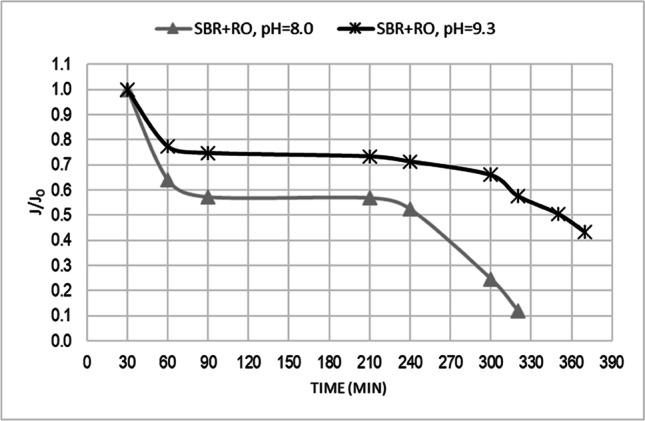
The normalized permeate flux (J/J_0_) at pH = 8.0 and pH = 9.3

Permeate flux is an important parameter in design and economical feasibility analysis of RO membrane separation process [7]. The average normalized flux (J/J_0_) was 0.53 and 0.68 respectively for process carried out in pH = 8.0 and pH = 9.3. (Fig. 2 and Table 3).

**Table 3:** The basic parameters of RO process carried out at pH = 8.0 and pH = 9.3

Parameter	RO pH = 8.0	RO pH = 9.3
max of J/J_0_	1.0	1.0
min of J/J_0_	0.12	0.43
average of J/J_0_	0.53	0.68
standard deviation	0.183	0.166

The observed decrease in permeate flow was lower for process conducted in pH = 9.3. Decrease in permeability during all filtration process was 0.88 for pH = 8.0 and 0.57 for pH = 9.3 (Fig. 2). The final flux at the end of RO process was 0.12 and 0.43 of initial flux, respectively for pH = 8.0 and pH = 9.3. The obtained results indicates that pH influences on permeate flux value. Table 3 summarize the results of reverse osmosis performance.

Observed during this study higher value of normalized permeate flux in pH = 9.3 is consistent with Trebouet et al. [43] findings, which state that permeate flux is lower at low pH. This pH-dependent behavior is attributed to the charge of the membrane and organic matter. Landfill leachate after biological pretreatment is still characterized by high value of hard-biodegradably organic matter, expressed as a COD. The NOM components contain a carboxyl and phenolic groups, which have a more negative charge at higher pH. At the same time, the surface charge of the membrane becomes more negative as the pH increases due to the deprotonation of the carboxylic groups and the protonation of the amine groups, which create the top layer of the polyamide RO membrane [45]. At higher pH electrostatic repulsions between the organic substances and membrane surface, as well as the increasing hydrophilicity of humic substances, decrease adsorption phenomena [43]. This results in reduction of organic fouling. Similar trends of decreased organic matter adsorption onto membrane surface at higher pH were observed by Hong and Elimelech [46], Chang et al. [47], Koc-Jurczyk and Jurczyk [48]. Increase of water flux with increasing pH value was also reported by Hoang et al. [32] and justified by expansion of the membrane matrix due to repulsion between like-charged dissociated functional groups on the pore walls. Bodzek and Płatkowska [49] have found that lower pH value influences on reduction in permeate flux, especially for solutions with high ions concentrations and increased amounts of divalent cations. They explain this by the fact, that calcium cations protonate the negatively charged functional groups of humic substances and form bridge connections between the negatively charged membrane surface and the negatively charged functional groups of humic substances. Lower pH and higher ionic strength promote adsorption and gel formation on the membrane surface due to the weakening of electrostatic repulsion between organic matter molecules [49].

Obtained results also showed two drops in permeate flux in time of filtration. The first one was observed during the first 60 min of filtration, in which permeate flux rapidly decreased to the 0.64 and 0.77 of initial flux, respectively for pH = 8.0 and pH = 9.3. The second flux decline started after 240 min of filtration and was not as rapid as the first one, but progressed successively with time. The first decrease can be associated with accumulation of dissolved substances, colloidal and suspended particles above the membrane surface (cake layer) and fouling on the membrane surface, resulted from an intermediate blocking. According to Chang et al. [47] fouling process is rapid for intermediate blocking, since fewer particles are required. Thus intermediate blocking is responsible for the fast flux decline at the beginning of membrane filtration. The second decrease in permeate flux is a consequence of standard blocking (adsorption and deposition of particles inside the pores, thus reducing pore volume) and scaling phenomena. The concentration of the feed subjected to the RO system increases because of its recirculation resulting in increase in the osmotic pressure of the solution on membrane [50]. As the filtration process is carried, the salt concentration on membrane surface is getting higher, creating concentration gradient in a boundary layer between the bulk solution and the membrane surface. This phenomenon of concentration polarization influences on a larger salt concentration difference across the membrane and leads to the solubility limit being exceeded, which results in a greater amount of scale deposition. This process occurs steadily as the amount of deposited particles increases and causes a gradually flux decline.

It is worth note, that according to Trebouet et al. [43] and Chang et al. [47], fouling at the membrane surface is attributed mainly to reversible phenomena, while pore adsorption and plugging by organic and inorganic compounds – to irreversible one.

- Effect of the feed pH on EC rejection

**Fig. 3:**
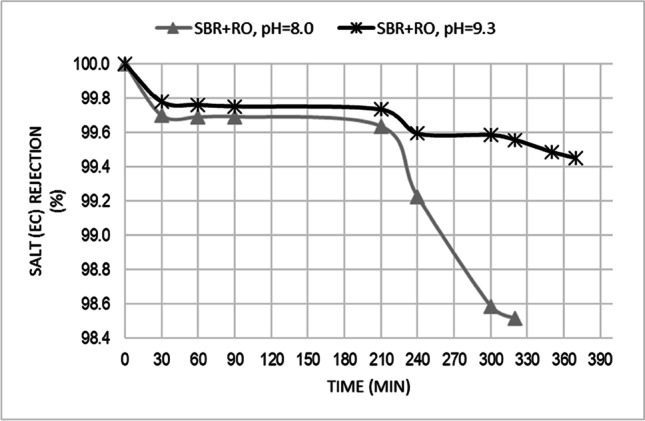
Salt (expressed as a EC) rejection at pH = 8.0 and pH = 9.3

The average salt (expressed as a EC) rejection was 99.4% in RO process carried out at pH = 8.0 and 99.7% at pH = 9.3.The final value of EC reduction at the end of the filtration procedure was 98.5 and 99.5% for pH = 8.0 and pH = 9.3, respectively (Fig. 3).

The above results complies the Hoang et al. [32] findings, which says that high values of chloride rejection are achieved at high pH. This behavior can be explained by the charge properties of the membrane. The membrane is negatively charged at high pH due to the deprotonation of carboxylic groups and adsorption of hydroxide ions on the membrane surface. The negative charge repulses chloride ions resulting in a high chloride rejection. The sodium rejection is also high to maintain the charge balance of the components in the solution [32]. The similar results were also achieved by Hong and Elimelech [46] who observed that TDS rejection increases with increasing pH.

**Fig. 4:**
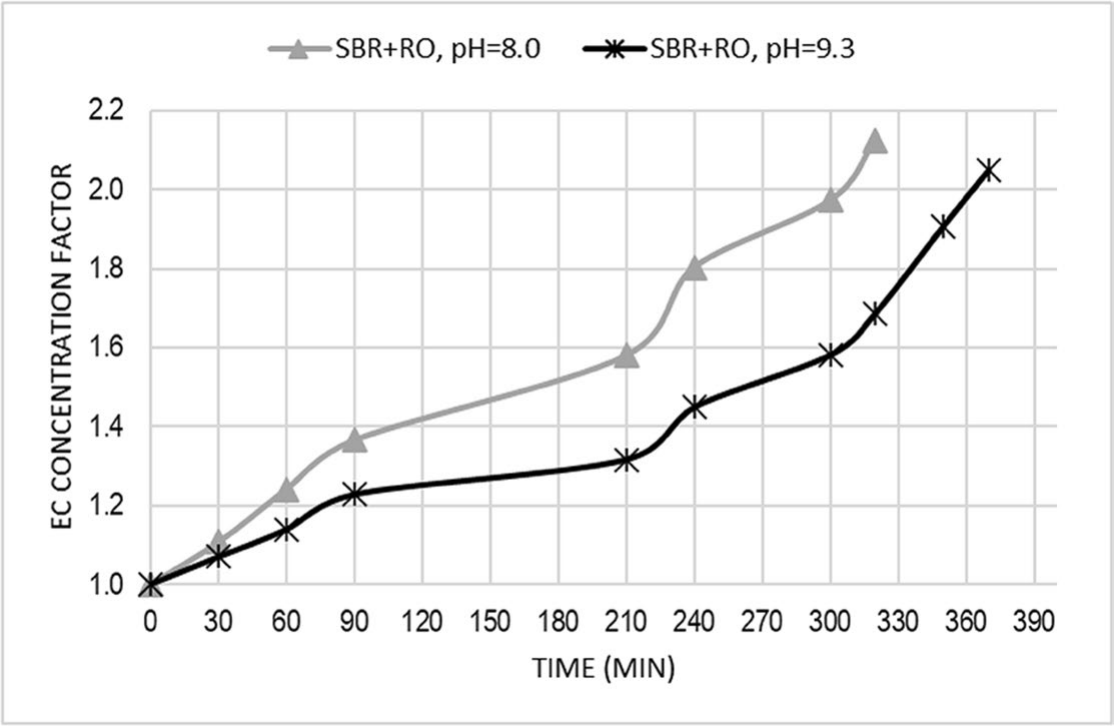
EC concentration factor at pH = 8.0 and pH = 9.3

The obtained EC concentration factors were similar for both pH value and were 2.13 and 2.05, respectively for pH value of 8.0 and 9.3 (Fig. 4). Ionic strength of the feed solution may significantly affect the solute rejection efficiency, because ionic strength affects the membrane surface zeta potential and in turn the electrostatic effect for solute rejection [51]. The recovery rate for caried out RO processes was set at 60% As the percentage recovery increased from 0 to 60%, and feedwater pressure remained constant, the salts in feed became more concentrated. This caused permeate flux and salt rejection to decrease what were observed during conducted experiment.

- Changes of feed pH

**Fig. 5:**
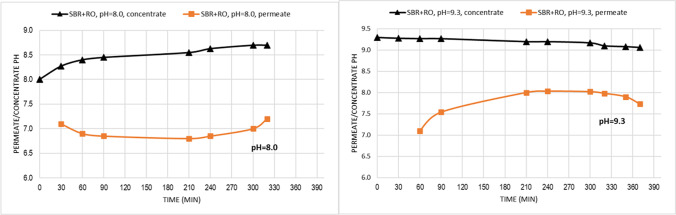
The permeate/concentrate at pH = 8.0 and pH = 9.3

With RO started at pH = 8, the pH of the permeate during filtration process was between 6.8 and 7.2 while the pH of the concentrate (feed) was between 8 and 8.7. The average pH of permeate was 7.0. At the RO process starting with pH of 9.3, the permeate pH fluctuations were wider—from 7.0 to 8.2—and the pH of concentrate (feed) was more stable: from 9.0 to 9.3. The average value of permeate pH was 7.8. In the two analyzed cases, the obtained permeate pH after RO process was lower than the feed pH (Fig. 5).

According to the Qin et al. [52] for each membrane there is a critical feed pH, above which RO permeate pH is lower than feed pH. The obtained results indicated that both variant of RO process conducted with pH = 8.0 and pH = 9.3 were carried above the critical feed pH. Qin et al. [52] noticed that for many membranes critical feed pH is within 4.3 ÷ 4.5 and is independent of their isoelectric points. Existence of HCO_3_^−^ in the feed and the transmissions of HCO_3_^−^ and protons in the RO process may play key roles to affect the permeate pH and dominate the critical feed pH [52]. When the feed pH is higher than 4.3, the dominant HCO_3_^−^ tends to be rejected by a RO membrane, resulting in lower [HCO_3_^−^] in permeate than that in feed. But the neutral carbon dioxide gas (or carbonic acid) passes through the RO membrane to provide proton and bicarbonate anion in permeate, thus reducing the permeate pH [52].

The phenomenon of lower permeate pH than the feed pH, at RO conducted (started) at pH above 7, was also analyzed by Park and Kwon [45] who reported that the shift in the permeate pH is not dominantly affected by the surface charge, hydrophilicity, and roughness of the membrane. According to them the shift in pH for the solutions passing through the RO membrane is a result of water dissociation in a permeate side to maintain the charge balance and water dissociation constant during filtration [45]. At high feed pH, the dissociation of water molecules in the permeate water intend to adjust the water dissociation constant to K_w_ = 1.0·10^–14^, thus inducing a decrease in the hydroxide concentration and an increase in the proton concentration relative to those of the feed solution [45]. As a result, the permeate pH decreases to less than that of the feed pH.

- Membrane fouling

The cleaning of membrane module was caried out after each RO process. After cleaning process, the permeate flux of cleaned membrane was tested. The degree of initial flux recovery was calculated as a ratio of permeate flux after cleaning procedure and permeate flux before RO process, both carried out in deionized water. The degree of initial flux recovery is presented in Table 4.

**Table 4:** The degree of initial flux recovery after membrane cleaning

Parametr	membrane after RO process at pH = 8.0	membrane after RO process at pH = 9.3
recovered permeability	0.68	0.61

The recovered permeability was 68% for membrane, through which the leachate at pH = 8.0 was filtered and 61% for membrane, through which the leachate at pH = 9.3 was processed. Because cleaning procedure was conducted with alkaline product, it was effective in removing organic fouling, biofouling and colloidal fouling. This indicate that inorganic deposition was responsible for the remaining pore blockade. Inorganic fouling (scaling) was responsible for 32% permeability decrease at pH = 8.0 and 39% permeability at pH = 9.3.

The higher scaling deposition was characteristic for higher pH value, what is in line with studies carried out by Bystrianský et al. [53], Jiang et al. [54], Park and Kwon [45], Matin et al. [28], Ashfaq et al. [29] and many others. Inorganic scaling is mainly caused by calcium carbonates, calcium sulfates, silicates and iron [29, 53, 55, 56]. The concentration of calcium, iron and silica were not determined in analyzed leachate before the RO process. Nevertheless the high value of EC indicate that these ions were presented in leachate. Due to 60% recovery in RO process, the concentration of sparingly soluble inorganic salts increased above the saturation level causing membrane scaling. According to Bystrianský et al. [53] the pH does not affect calcium sulphate crystallization, however it has an impact on iron crystallization. The most intensive membrane scaling with iron (Fe^3+^) occurs in alkaline medium. Moreover, the presence of Fe^3+^ ions increases fouling due to complex formation between iron and natural organic matter that may precipitate on the membrane surface or inside the pores [57].. The solubility of calcium carbonate and silicates is pH-dependent and RO operation at higher pH increases the likelihood of their precipitation. According to Peña et al. [55] inorganic scaling is in about 30% responsible for membrane blocking at neutral pH. At conducted research this value was higher: 32% and 39% for pH = 8.0 and pH = 9.3, respectively. The remained over 60% was the organic and colloidal fouling which was removed by alkaline cleaning.

In landfill leachate RO filtration effluent organic matter (EfOM) containing large fraction of soluble microbial products has been identified as a major organic foulant of membrane processes [58, 59]. It is also reported, that hydrophilic fraction of EfOM comprised of polysaccharide-like and protein-like functional groups as well as colloidal materials play role in high-pressure filtration. Formation an initial fouling layer of the RO membranes can be attributed to adsorption of the uncharged or oppositely charged polysaccharide-like and protein-like substances onto RO membrane surfaces [59]. In conducted analyses organic fouling was in 68% responsible for membrane blocking at pH = 8.0 and in 61% for membrane blocking at pH = 9.3. These values are lower than obtained in Peña et al. [55] studies, which report the value of 70% responsible for organic fouling at neutral pH. This confirms the effectiveness of using higher pH values to reduce organic fouling.

### Reverse osmosis efficiency

The obtained removal rates in RO process carried out in pH = 8.0 and pH = 9.3 are presented in Table 5

**Table 5:** Removal rates in RO process carried out in pH = 8.0 and pH = 9.3

Parameter	Inlet* (after SBR pretreatment) pH 8.0/9.3	Removal rate ± st. dev. after RO treatment pH = 8.0 (%)	Removal rate ± st. dev. after RO treatment pH = 9.3 (%)
pH	8.0/9.3	pH = 7.0	pH = 7.8
EC	12.9/11.25	99.1 ± 0.6	99.6 ± 0.6
TDS	11,520/10666	98.1 ± 0.7	99. 5 ± 0.7
COD	2510	99.7 ± 0.5	99.0 ± 0.5
BOD	19	99.8 ± 0.1	99.9 ± 0.1
N-NH_4_^+^	42	99.3 ± 0.3	95.5 ± 0.3
B	12.2	89.0 ± 1.2	95.0 ± 1.2
Cl^−^	n.a./2200	98.5 ± 0.1	99.5 ± 0.1

*All in mg/L apart EC (mS/cm) and pH, n.a. -not analyzed, st.dev-standard deviation.

The obtained permeate pH after RO process was lower than the feed pH. As it was explained earlier, the shift in pH for the solution passing through RO membrane can be the result of water dissociation in a permeate side to maintain the charge balance and water dissociation constant during filtration [45].

The COD retention was slightly affected by pH and was 99.7% at pH = 8.0 and 99% at pH = 9.3. Similar results were obtained by Trebouet et al. [43], who reported that weight distribution study indicates the majority of compounds accountable for COD is below 1000 Da. This fraction comprises mainly fulvic acids and the high-pressure filtration process is efficient in treatment of such a kind of refractory organic matter. There was no difference in BOD removal during RO filtration since the most organic substances expressed as BOD were removed during SBR pretreatment with efficiency of 97.3%.

There was observed that TDS rejection was higher at higher pH value, what is in line with Hong and Elimelech findings [46]. These observations can be explained by so-called Donnan exclusion mechanism of charged porous membrane [46].

A difference in removal efficiency was observed for chloride, whose removal rate at pH = 9.3 (99.5%) was slightly higher than at pH = 8.0 (98.5%). It is in line with Hoan et al. [32] findings, that at higher pH value, the higher negative charge of membrane repulses chloride ions resulting in a high chloride rejection. The similar phenomenon was observed in case of TDS.

The more significant difference in removal rates was observed for nitrogen ammonia and boron. The efficiency of nitrogen ammonia rejection was 99.3% for pH = 8.0 and 95.5% for pH = 9.3. Ammonium which has pKa of 9.25, changes to uncharged ammonia at higher pH. Therefore, ammonium ion rejection decreases at a higher pH due to decrease in the hydration radius of the uncharged ammonia [45]. The boron removal rates were 89% at pH = 8.0 and 95% at pH = 9.3. Dissociation of B(OH)_3_ to borate B(OH)_4_ occurred at pKa = 9.27. This means that in aqueous solution at pH below 9.27 boron exists as an uncharged B(OH)_3_, and in solution above pH 9.27 – as a B(OH)_4_^−^. The neutral species are easily transported through the membrane due both to lack of steric hindrance as well as lack of charge repulsion. It is possible that a slightly higher reduction of EC than monovalent ions (N-NH_4_^+^, Cl^−^) at pH = 9.3 is the result of Donnan exclusion theory. Large, more strongly charged ions are strongly rejected than smaller ones like N-NH_4_^+^, Cl^−^. Nevertheless, at pH = 8.0 no such regularity was observed. Thus, drawing definitive conclusions requires additional, more extensive studies and experiments.

## Conclusions

The aim of this paper was to performance analysis of integrated SBR + RO system for leachate treatment at two variants: with pH = 9.3 and pH = 8.0. The landfill leachate at intermediate stage was taken into analyses.

After SBR pretreatment leachate characterized with good removal efficiency of N-NO_3_^−^, BOD and N-NH_4_^+^. The removal ratios for EC, COD, Cl^−^ and B were lower: 37.2%, 32.5%, 11.6% and 3.9%, respectively.

Biologically pretreated leachate was directed into reverse osmosis system. In RO process for landfill leachate, the normalized permeate flux was higher at pH = 9.3 than at pH = 8.0. Observed decreased membrane fouling at higher pH value can be explained by electrostatic repulsion between the negatively charged membrane surface and organic substances. A two-step membrane fouling was observed, independently of the process pH. At first step fouling at the membrane surface was prevalent, and it was caused mainly by organic pollutants. Pore adsorption and deposition by inorganic compounds dominated at second one. The obtained permeate pH after RO process was lower than the feed pH in two analyzed variants of RO process.

The highest differences in removal rates during RO process were observed for boron, which had a higher removal efficiency at pH of 9.3 compared to pH = 8, and ammonia nitrogen, whose removal rate decreased at pH = 9.3. A slightly higher values of salt rejection, expressed as a EC, were achieved at pH = 9.3.

Results of washing procedure has showed that organic fouling was in 68% responsible for membrane blocking at pH = 8.0 and in 61% for membrane blocking at pH = 9.3. It confirms that that the organic matter content should be taken into account when determining the operating parameters (pH values) of the RO system. The proper operating parameters may also have business implications in terms of increase productivity and permeate quality, decreasing operating cost, additional pretreatment as well as in membrane lifetime increase. Nevertheless, fouling process on membrane surface is very complicated and influenced by many factors, therefore a more detailed studies of membrane fouling are required to determine the complex nature of this phenomenon.

## Data Availability

The datasets used and/or analyzed during the current study are available from the corresponding author on reasonable request.
